# Therapeutic trial in hereditary angioedema type III: Icatibant

**DOI:** 10.1186/1939-4551-8-S1-A220

**Published:** 2015-04-08

**Authors:** Laila Sabino Garro, Maria Helena Mattos Porter, Fernanda Komaroff, Caroline Terumi Adachi, Maria Teresinha Malheiros, Yara Mello, Marisa Rosimeire Ribeiro

**Affiliations:** 1 Edmundo Vasconcelos Hospital, Brazil

## Background

Hereditary angioedema (HAE) is a disease transmitted by autosomal dominant inheritance, characterized by quantitative and / or functional deficiency of C1 inhibitor (C1-INH), which causes episodes of swelling, with involvement of many organs. HAE is currently divided into three groups. The HAE type III is a less frequent disorder that mainly affects women and is characterized by normal levels and activity of C1-INH.

## Methods

Literature review and case description.

## Results

We assessed a 33 years old female with episodes of lip angioedema and facial edema since March 2014, with no pruritus, with paresthesia, sometimes associated with suffocation, lasting up to 5 days despite of the use of corticosteroids and antihistamines. Patient remained with attacks twice a week with edema in different regions of the body. She did not use inhibitors of angiotensin converting enzyme, but she used contraceptive containing estrogen. The causes of allergic or acquired angioedema were ruled out. Laboratory investigation showed normal results for inhibitor quantitative and functional C1 and normal levels of C4 and C1q. The change of the oral contraceptive to another free of estrogen did not bring remission of episodes of edema. In June 2014 the patient was seen in the emergency room with dysphonia and suffocation. The patient was submitted to the standard treatment for anaphylaxis and a subcutaneous dose of Icatibant 30 mg. She had significant improvement of the symptoms in less than 1 hour. After that episode we decided to treat her with Danazol 200mg daily. Since then this patient had no more symptoms.

## Conclusions

We report a case of hereditary angioedema type III who received Icatibant as a therapeutic trial because of an acute laryngeal edema. In HAE type III laboratory investigation is normal and clinical evidence becomes critical to the diagnosis. In this case, the immediate response to the use of the bradykinin receptor inhibitor reinforced the clinical evidence and led to the early diagnosis of hereditary angioedema type III.

## Consent

Written informed consent was obtained from the patient for publication of this abstract and any accompanying images. A copy of the written consent is available for review by the Editor of this journal.

